# Gradient estimates and Liouville-type theorems for a weighted nonlinear elliptic equation

**DOI:** 10.1186/s13660-018-1705-z

**Published:** 2018-05-10

**Authors:** Bingqing Ma, Yongli Dong

**Affiliations:** 1 College of Physics and Materials Science, Henan Normal University, Xinxiang, P.R. China; 2 Department of Mathematics, Henan Normal University, Xinxiang, P.R. China

**Keywords:** 58J35, 35B45, Gradient estimate, Nonlinear elliptic equation, Liouville-type theorem

## Abstract

We consider gradient estimates for positive solutions to the following nonlinear elliptic equation on a smooth metric measure space $(M, g,e^{-f}\,dv)$:

$$\Delta_{f} u+au\log u+bu=0, $$

where *a*, *b* are two real constants. When the ∞-Bakry–Émery Ricci curvature is bounded from below, we obtain a global gradient estimate which is not dependent on $|\nabla f|$. In particular, we find that any bounded positive solution of the above equation must be constant under some suitable assumptions.

## Introduction

Let $(M, g)$ be an *n*-dimensional complete Riemannian manifold and *f* be a smooth function defined on *M*. Then the triple $(M, g,e^{-f}\, dv)$ is called a smooth metric measure space, where *dv* denotes the volume element of the metric *g* and $e^{-f}\,dv$ is called the weighted measure. On the smooth metric measure space $(M, g,e^{-f}\, dv)$, the *m*-Bakry–Émery Ricci curvature (see [1–3]) is defined by

1.1 $$ \mathrm{Ric}_{f}^{m}=\mathrm{Ric}+ \nabla^{2}f-\frac{1}{m-n}\,df\otimes df, $$

where $m\geq n$ is a constant, and $m=n$ if and only if *f* is a constant. We define

1.2 $$ \mathrm{Ric}_{f}=\mathrm{Ric}+\nabla^{2}f. $$

Then $\mathrm{Ric}_{f}$ can be seen as the ∞-dimensional Bakry–Émery Ricci curvature. However, there are many differences between the *m*-Bakry–Émery Ricci curvature and the ∞-Bakry–Émery Ricci curvature. For example, there exist complete noncompact Riemannian manifolds which satisfy $\mathrm{Ric}_{f}=\lambda g$ for some positive constant *λ* (which is called the shrinking gradient Ricci soliton), but not for $\mathrm{Ric}_{f}^{m}=\lambda g$. We recall that the *f*-Laplacian $\Delta_{f}$ on $(M, g,e^{-f}\,dv)$ is defined by

$$\Delta_{f}=\Delta-\nabla f\nabla. $$

Since we have the Bochner formula with respect to *f*-Laplacian:

$$\frac{1}{2}\Delta_{f}|\nabla u|^{2}\geq \frac{1}{m}(\Delta_{f}u)^{2}+\nabla u\nabla( \Delta_{f}u)+\mathrm{Ric}_{f}^{m}(\nabla u,\nabla u), $$

which is similar to the Bochner formula associated with the Laplacian, many results with respect to the Laplacian have been generalized to those of the *f*-Laplacian under the *m*-dimensional Bakry–Émery Ricci curvature. For example, see [4–7] and the references therein. But for elliptic gradient estimates for *f*-Laplacian under the ∞-Bakry–Émery Ricci curvature, in order to using the weighted comparison theorem, the assumption $|\nabla f|\leq\theta$ is forced commonly.

In this paper, under the assumption that the ∞-Bakry–Émery Ricci curvature is bounded from below, we consider the following nonlinear elliptic equation:

1.3 $$ \Delta_{f}u+au\log u+bu=0, $$

where *a*, *b* are two real constants. Inspired by the ideas of Brighton in [8], we can obtain global gradient estimates for positive solutions to (1.3) without any restriction on $|\nabla f|$.

### Theorem 1.1

*Let* $(M, g,e^{-f}\,dv)$ *be an* *n*-*dimensional complete smooth metric measure space with* $\mathrm{Ric}_{f}(B_{p}(2R))\geq-(n-1)K$, *where* $K\geq0$ *is a constant*. *Suppose that* *u* *is a positive solution to* (1.3) *with* $u\leq A$ *on* $B_{p}(2R)$. *Then on* $B_{p}(R)$ *with* $R>1$, *the following inequality holds*:

1.4 $$ \begin{aligned} |\nabla u|^{2}\leq C A^{2} \biggl[\max \biggl\{ \frac{4}{5}b+a \biggl(1+ \frac {4}{5}L \biggr),0 \biggr\} +K+\frac{|\beta|+1}{R} \biggr], \end{aligned} $$

*where* *C* *is a positive constant which depends on the dimension* *n*, $\beta=\max_{\{x|d(x,p)=1\}} \Delta_{f} r(x)$ *and*

1.5 $$ L= \textstyle\begin{cases} \sup_{B_{p}(2R)}(\log u), &\textit{if } a\geq0, \\ \inf_{B_{p}(2R)}(\log u), &\textit{if } a< 0. \end{cases} $$

Letting $R\rightarrow\infty$ in (1.4), we obtain the following global estimates on complete noncompact Riemannian manifolds:

### Corollary 1.2

*Let* $(M, g,e^{-f}\,dv)$ *be an* *n*-*dimensional complete smooth metric measure space with* $\mathrm{Ric}_{f} \geq-(n-1)K$, *where* $K\geq0$ *is a constant*. *If* *u* *is a positive solution to* (1.3) *with* $u\leq A$, *then we have*

1.6 $$ \begin{aligned} |\nabla u|^{2}\leq C A^{2} \biggl[\max \biggl\{ \frac{4}{5}b+a \biggl(1+ \frac {4}{5}L \biggr),0 \biggr\} +K \biggr], \end{aligned} $$

*where*

1.7 $$ L= \textstyle\begin{cases} \sup_{M}(\log u), &\textit{if } a\geq0, \\ \inf_{M}(\log u), &\textit{if } a< 0. \end{cases} $$

Using the ideas of the proof of Theorem 1.1, by choosing $\tilde{h}=\log u$ a gap develops between the constants, and we also establish the following.

### Theorem 1.3

*Let* $(M, g,e^{-f}\,dv)$ *be an* *n*-*dimensional complete smooth metric measure space with* $\mathrm{Ric}_{f}(B_{p}(2R))\geq-(n-1)K$, *where* $K\geq0$ *is a constant*. *Suppose that* *u* *is a positive solution to* (1.3) *on* $B_{p}(2R)$ *such that*:

1. *either* $\nabla f \nabla(\log u)-a\log u-b\leq\delta|\nabla (\log u)|^{2}$ *for some* $0\leq\delta<\frac{1}{2}$;
2. *or* $\nabla f \nabla(\log u)-a\log u-b\geq2 |\nabla(\log u)|^{2}$.

*Then on* $B_{p}(R)$ *with* $R>1$, *the following inequality holds*:

1.8 $$ \big|\nabla(\log u)\big|^{2}\leq\frac{C_{1}(n,\delta,\beta)}{R}+C_{2}(n, \delta)\max\bigl\{ a+(n-1)K,0\bigr\} , $$

*where* $\beta=\max_{\{x|d(x,p)=1\}}\Delta_{f}r(x)$.

Letting $R\rightarrow\infty$ in (1.8), we obtain the following global estimates on complete noncompact Riemannian manifolds:

### Corollary 1.4

*Let* $(M, g,e^{-f}\,dv)$ *be an* *n*-*dimensional complete smooth metric measure space with* $\mathrm{Ric}_{f} \geq-(n-1)K$, *where* $K\geq0$ *is a constant*. *Let* *u* *be a positive solution to* (1.3). *Then under the assumption of either* (1) *or* (2) *as in Theorem* 1.3, *we have*

1.9 $$ \begin{aligned} \big|\nabla(\log u)\big|^{2}\leq C(n, \delta)\max\bigl\{ a+(n-1)K,0\bigr\} . \end{aligned} $$

Clearly, if either $u\leq e^{-(\frac{5}{4}+\frac{b}{a})}$ and $a>0$, or $u\geq e^{-(\frac{5}{4}+\frac{b}{a})}$ and $a<0$, then we have $\frac {4}{5}b+ a (1+\frac{4}{5}L )\leq0$. This gives the following result.

### Corollary 1.5

*Let* $(M, g,e^{-f}\,dv)$ *be an* *n*-*dimensional complete smooth metric measure space with* $\mathrm{Ric}_{f} \geq0$.

1. *There exists no bounded positive solution to* (1.3) *with* $a>0$ *and* $u\leq e^{-(\frac{5}{4}+\frac{b}{a})}$;
2. *if* $a<0$ *and* $u\geq e^{-(\frac{5}{4}+\frac{b}{a})}$, *then any bounded positive solution to* (1.3) *must be constant* $u=e^{-\frac{b}{a}}$.

### Remark 1.1

In particular, when $a=0$, Eq. (1.3) becomes

1.10 $$ \Delta_{f}u+bu=0 $$

and (1.6) becomes

1.11 $$ \begin{aligned} |\nabla u|^{2}\leq C A^{2} \biggl[\max \biggl\{ \frac{4}{5}b,0 \biggr\} +K \biggr]. \end{aligned} $$

In this case, on a complete smooth metric measure space $(M, g,e^{-f}\, dv)$ with $\mathrm{Ric}_{f} \geq0$, there exists no bounded positive solution to (1.10) with $b<0$. On the other hand, if $a=b=0$, our Theorem 1.1 becomes Theorem 1 of Brighton in [8].

### Remark 1.2

It is easy to see from Corollary 1.4 that if *u* is a positive solution to (1.3) with $a\leq-(n-1)K$ satisfying either (1) or (2) in Theorem 1.3, then $u=e^{-\frac{b}{a}}$ is a constant. In particular, if $a=b=0$, then our Theorem 1.3 becomes Theorem 3 of Brighton in [8].

### Remark 1.3

Some related results for gradient estimates of positive solutions to

1.12 $$ \Delta_{f}u+au\log u=0 $$

can be found in [9–11]. Moreover, Qian in [10] used a different method to derive similar estimates to (1.12) with constant *f*. On the other hand, if we assume $\mathrm{Ric}_{f} \geq -(n-1)K$ and $|\nabla f|\leq\theta$, then from (1.1), we obtain

$$\begin{aligned}\mathrm{Ric}_{f}^{m}&= \mathrm{Ric}_{f}-\frac{1}{m-n}\, df\otimes df \\ &\geq-(n-1) \biggl(K+\frac{\theta^{2}}{(m-n)(n-1)} \biggr):=-(n-1)\tilde{K}. \end{aligned} $$

Hence, Theorem 1.5 in [11] follows from Theorem 1.1 of [11] immediately. However, our estimates in this paper are not dependent on $|\nabla f|$.

## Proof of results

We firstly give the following lemma which plays an important role in the proof of main results.

### Lemma 2.1

*Let* *u* *be a positive solution to* (1.3) *with* $u\leq A$ *and* $\mathrm{Ric}_{f}\geq-(n-1)K$ *for some positive constant* *K*. *Denote* $\tilde{u}=u/A$ *and* $h=\tilde {u}^{\epsilon}$ *for* $\epsilon\in(0,1)$. *If there exists one positive constant* *δ* *satisfying*

2.1 $$ \frac{1}{n}+\frac{2(\epsilon-1)}{n\epsilon\delta}\geq0, $$

*then we have*

2.2 $$ \begin{aligned}[b] \frac{1}{2}\Delta_{f}| \nabla h|^{2}\geq{}& \biggl(\frac{(\epsilon-1)^{2}}{n\epsilon^{2}}-\frac{\epsilon -1}{\epsilon}+ \frac{2\delta(\epsilon-1)}{n\epsilon} \biggr)\frac{|\nabla h|^{4}}{h^{2}}+\frac{\epsilon-1}{\epsilon }\frac{\nabla h}{h} \nabla\bigl(|\nabla h|^{2}\bigr) \\ &-\bigl[a+\tilde{b}\epsilon+(n-1)K+a\tilde{L}\bigr]|\nabla h|^{2}, \end{aligned} $$

*where*

2.3 $$ \tilde{L}= \textstyle\begin{cases} \sup_{M}(\log h), &\textit{if } a\geq0, \\ \inf_{M}(\log h), &\textit{if } a< 0. \end{cases} $$

### Proof

Under the scaling $u\rightarrow\tilde{u}=u/A$, it follows from (1.3) that *ũ* satisfies

2.4 $$ \Delta_{f} \tilde{u}+a\tilde{u}\log \tilde{u}+\tilde{b} \tilde{u}=0, $$

where the constant *b̃* is given by $\tilde{b}=b+a\log A$. Let $h=\tilde{u}^{\epsilon}$, where $\epsilon\in(0,1)$ is a constant to be determined. Then we have

2.5 $$ \log h=\epsilon\log\tilde{u}. $$

Since $0<\tilde{u}\leq1$, we have $\log h\leq0$ and

2.6 $$ \begin{aligned}[b] \Delta_{f} h&= \Delta_{f}\bigl(\tilde{u}^{\epsilon}\bigr)=\epsilon(\epsilon-1) \tilde {u}^{\epsilon-2}|\nabla \tilde{u}|^{2}+\epsilon \tilde{u}^{\epsilon-1}\Delta_{f} \tilde{u} \\ &=\epsilon(\epsilon-1)\tilde{u}^{\epsilon-2}|\nabla\tilde {u}|^{2}-a \epsilon\tilde{u}^{\epsilon}\log\tilde{u}-\tilde{b}\epsilon \tilde{u}^{\epsilon}\\ &=\frac{\epsilon-1}{\epsilon} \frac{|\nabla h|^{2}}{h}-ah\log h-\tilde {b}\epsilon h, \end{aligned} $$

which implies

2.7 $$ \begin{aligned}[b] \nabla h\nabla\Delta_{f} h&= \nabla h\nabla \biggl(\frac{\epsilon-1}{\epsilon} \frac{|\nabla h|^{2}}{h}-ah\log h-\tilde{b} \epsilon h \biggr) \\ &=\frac{\epsilon-1}{\epsilon}\nabla h\nabla\frac{|\nabla h|^{2}}{h}-a\nabla h\nabla(h\log h)- \tilde{b}\epsilon|\nabla h|^{2} \\ &=\frac{\epsilon-1}{\epsilon}\frac{\nabla h}{h}\nabla\bigl(|\nabla h|^{2} \bigr)-\frac{\epsilon-1}{\epsilon}\frac{|\nabla h|^{4}}{h^{2}} \\ &\quad{}-ah\log h\frac{|\nabla h|^{2}}{h}-(a+\tilde{b}\epsilon)|\nabla h|^{2}. \end{aligned} $$

Thus, under the assumption $\mathrm{Ric}_{f}\geq-(n-1)K$, one has

2.8 $$ \begin{aligned}[b] \frac{1}{2}\Delta_{f}| \nabla h|^{2}&=|\nabla^{2}h|^{2}+\nabla h\nabla \Delta_{f} h+\mathrm{Ric}_{f} (\nabla h,\nabla h) \\ &\geq\frac{1}{n}(\Delta h)^{2}+\nabla h\nabla \Delta_{f} h-(n-1)K|\nabla h|^{2} \\ &=\frac{1}{n} \biggl(\frac{\epsilon-1}{\epsilon} \frac{|\nabla h|^{2}}{h}+\nabla f\nabla h-ah\log h-\tilde{b}\epsilon h \biggr)^{2}+\frac{\epsilon-1}{\epsilon } \frac{\nabla h}{h}\nabla \bigl(|\nabla h|^{2}\bigr) \\ &\quad{}-\frac{\epsilon-1}{\epsilon}\frac{|\nabla h|^{4}}{h^{2}} -(ah\log h)\frac{|\nabla h|^{2}}{h}-\bigl[a+ \tilde{b}\epsilon+(n-1)K\bigr]|\nabla h|^{2} \\ &= \biggl(\frac{(\epsilon-1)^{2}}{n\epsilon^{2}}-\frac{\epsilon-1}{\epsilon } \biggr)\frac{|\nabla h|^{4}}{h^{2}} + \frac{2(\epsilon-1)}{n\epsilon}\frac{|\nabla h|^{2}}{h} (\nabla f\nabla h-ah\log h-\tilde{b}\epsilon h ) \\ &\quad{}+\frac{1}{n} (\nabla f\nabla h-ah\log h-\tilde{b}\epsilon h )^{2}+\frac{\epsilon-1}{\epsilon }\frac{\nabla h}{h}\nabla\bigl(|\nabla h|^{2}\bigr) \\ &\quad{}-\bigl[a+\tilde{b}\epsilon+(n-1)K+a\log h\bigr]|\nabla h|^{2}. \end{aligned} $$

For any fixed point *p*, if there exists a positive constant *δ* such that $\nabla f\nabla h-ah\log h-\tilde{b}\epsilon h\leq \delta\frac{|\nabla h|^{2}}{h}$, then from (2.8), we can deduce

2.9 $$\begin{aligned} \frac{1}{2}\Delta_{f}| \nabla h|^{2}\geq{}& \biggl(\frac{(\epsilon-1)^{2}}{n\epsilon ^{2}}-\frac{\epsilon-1}{\epsilon} \biggr) \frac{|\nabla h|^{4}}{h^{2}} +\frac{2(\epsilon-1)}{n\epsilon}\frac{|\nabla h|^{2}}{h} \biggl(\delta \frac {|\nabla h|^{2}}{h} \biggr) \\ &+\frac{1}{n} (\nabla f\nabla h-ah\log h-\tilde{b}\epsilon h )^{2}+\frac{\epsilon-1}{\epsilon }\frac{\nabla h}{h}\nabla\bigl(|\nabla h|^{2}\bigr) \\ &-\bigl[a+\tilde{b}\epsilon+(n-1)K+a\log h\bigr]|\nabla h|^{2} \\ \geq{}& \biggl(\frac{(\epsilon-1)^{2}}{n\epsilon^{2}}-\frac{\epsilon-1}{\epsilon }+\frac{2\delta(\epsilon-1)}{n\epsilon} \biggr) \frac{|\nabla h|^{4}}{h^{2}}+\frac{\epsilon-1}{\epsilon }\frac{\nabla h}{h}\nabla\bigl(|\nabla h|^{2}\bigr) \\ &-\bigl[a+\tilde{b}\epsilon+(n-1)K+a\tilde{L}\bigr]|\nabla h|^{2}. \end{aligned}$$

On the contrary, if $\nabla f\nabla h-ah\log h-\tilde{b}\epsilon h\geq \delta\frac{|\nabla h|^{2}}{h}$ at the point *p*, then from (2.8), we can deduce

2.10 $$ \begin{aligned}[b] \frac{1}{2}\Delta_{f}| \nabla h|^{2}\geq{}& \biggl(\frac{(\epsilon-1)^{2}}{n\epsilon ^{2}}-\frac{\epsilon-1}{\epsilon} \biggr) \frac{|\nabla h|^{4}}{h^{2}} +\frac{2(\epsilon-1)}{n\epsilon\delta} (\nabla f\nabla h-ah\log h-\tilde{b} \epsilon h )^{2} \\ &+\frac{1}{n} (\nabla f\nabla h-ah\log h-\tilde{b}\epsilon h )^{2}+\frac{\epsilon-1}{\epsilon }\frac{\nabla h}{h}\nabla\bigl(|\nabla h|^{2}\bigr) \\ &-\bigl[a+\tilde{b}\epsilon+(n-1)K+a\log h\bigr]|\nabla h|^{2} \\ ={}& \biggl(\frac{(\epsilon-1)^{2}}{n\epsilon^{2}}-\frac{\epsilon-1}{\epsilon } \biggr)\frac{|\nabla h|^{4}}{h^{2}} + \biggl(\frac{1}{n}+\frac{2(\epsilon-1)}{n\epsilon\delta} \biggr) (\nabla f\nabla h-ah\log h- \tilde{b}\epsilon h )^{2} \\ &+\frac{\epsilon-1}{\epsilon }\frac{\nabla h}{h}\nabla\bigl(|\nabla h|^{2} \bigr)-\bigl[a+\tilde{b}\epsilon +(n-1)K+a\log h\bigr]|\nabla h|^{2} \\ \geq{}& \biggl[\frac{(\epsilon-1)^{2}}{n\epsilon^{2}}-\frac{\epsilon-1}{\epsilon} + \biggl(\frac{1}{n}+ \frac{2(\epsilon-1)}{n\epsilon\delta} \biggr)\delta^{2} \biggr]\frac{|\nabla h|^{4}}{h^{2}}+ \frac{\epsilon-1}{\epsilon }\frac{\nabla h}{h}\nabla\bigl(|\nabla h|^{2}\bigr) \\ &-\bigl[a+\tilde{b}\epsilon+(n-1)K+a\log h\bigr]|\nabla h|^{2} \\ \geq{}& \biggl(\frac{(\epsilon-1)^{2}}{n\epsilon^{2}}-\frac{\epsilon-1}{\epsilon }+\frac{2\delta(\epsilon-1)}{n\epsilon} \biggr) \frac{|\nabla h|^{4}}{h^{2}}+\frac{\epsilon-1}{\epsilon }\frac{\nabla h}{h}\nabla\bigl(|\nabla h|^{2}\bigr) \\ &-\bigl[a+\tilde{b}\epsilon+(n-1)K+a\tilde{L}\bigr]|\nabla h|^{2} \end{aligned} $$

as long as (2.1) holds.

Therefore, in these two cases the estimate (2.2) holds, which finishes the proof of the Lemma 2.1. □

### Proof of Theorem 1.1

In order to obtain the upper bound of $|\nabla h|$ by using the maximum principle for (2.2), we need to choose *ϵ*, *δ* such that the coefficient of $\frac{|\nabla h|^{4}}{h^{2}}$ in (2.2) is positive. That is, we need

2.11 $$ \frac{(\epsilon-1)^{2}}{n\epsilon^{2}}-\frac{\epsilon-1}{\epsilon}+\frac {2\delta(\epsilon-1)}{n\epsilon}>0. $$

In particular, by choosing $\epsilon=\frac{4}{5}$ and letting $\delta\rightarrow\frac{1}{2}$, we find that the inequality (2.1) holds and (2.2) becomes

2.12 $$ \begin{aligned}[b] \frac{1}{2}\Delta_{f}| \nabla h|^{2}\geq{}&\frac{4n-3}{16n}\frac{|\nabla h|^{4}}{h^{2}}- \frac{1}{4}\frac {\nabla h}{h}\nabla\bigl(|\nabla h|^{2}\bigr) \\ &-\bigl[a+\tilde{b}\epsilon+(n-1)K+a\tilde{L}\bigr]|\nabla h|^{2}. \end{aligned} $$

As in [8], we define a cut-off function $\psi\in C^{2}([0,+\infty))$ by

2.13 $$ \psi(t)= \textstyle\begin{cases} 1, & t\in[0,R];\\ 0, & t\in[2R,+\infty], \end{cases} $$

satisfying $\psi(t)\in[0,1]$ and

2.14 $$ -\frac{C}{R}\leq\frac{\psi'(t)}{\sqrt{\psi}}\leq0,\qquad \big| \psi''(t)\big|\leq\frac {C}{R^{2}}, $$

where *C* is a positive constant. Let

$$\phi=\psi\bigl(d(x,p)\bigr). $$

Using Eq. (2.19) in [8] (see Eq. (4.5) in [5] or [12, Theorem 3.1]), we obtain

2.15 $$ \Delta_{f}\phi\geq-\frac{C\beta}{R}- \frac{C(n-1)K(2R-1)}{R}-\frac{C}{R^{2}} $$

and

2.16 $$ \frac{|\nabla\phi|^{2}}{\phi}\leq\frac{C}{R^{2}}. $$

Denote by $B_{p}(R)$ the geodesic ball centered at *p* with radius *R*. Let $G=\phi|\nabla h|^{2}$. Assume *G* achieves its maximum at the point $x_{0}\in B_{p}(2R)$ and assume $G(x_{0})>0$ (otherwise the proof is trivial). Then, at the point $x_{0}$,

$$\Delta_{f} G\leq0,\qquad \nabla\bigl(|\nabla h|^{2}\bigr)=- \frac{|\nabla h|^{2}}{\phi} \nabla\phi $$

and

2.17 $$ \begin{aligned} [b]0\geq{}& \Delta_{f}G \\ ={}&\phi\Delta_{f}\bigl(|\nabla h|^{2}\bigr)+|\nabla h|^{2}\Delta_{f}\phi+2\nabla\phi\nabla |\nabla h|^{2} \\ ={}&\phi\Delta_{f}\bigl(|\nabla h|^{2}\bigr)+ \frac{\Delta_{f}\phi}{\phi}G -2\frac{|\nabla\phi|^{2}}{\phi^{2}}G \\ \geq{}&\frac{\Delta_{f}\phi}{\phi}G -2\frac{|\nabla\phi|^{2}}{\phi^{2}}G+2\phi \biggl[\frac{4n-3}{16n} \frac{|\nabla h|^{4}}{h^{2}}-\frac{1}{4}\frac{\nabla h}{h}\nabla\bigl(|\nabla h|^{2}\bigr) \\ &-\bigl[a+\tilde{b}\epsilon+(n-1)K+a\tilde{L}\bigr]|\nabla h|^{2} \biggr] \\ ={}&\frac{\Delta_{f}\phi}{\phi}G -2\frac{|\nabla\phi|^{2}}{\phi^{2}}G+\frac {4n-3}{8n} \frac{G^{2}}{\phi h^{2}}+\frac{G}{2\phi}\nabla\phi\frac{\nabla h}{h} \\ &-2\bigl[a+\tilde{b}\epsilon+(n-1)K+a\tilde{L}\bigr]G, \end{aligned} $$

where in the second inequality, we used (2.12). Multiplying both sides of (2.17) by $\frac{\phi}{G}$, we obtain

2.18 $$ \begin{aligned}[b] \frac{4n-3}{8n}\frac{G}{h^{2}} \leq{}&{-}\frac{1}{2}\nabla\phi\frac{\nabla h}{h}+2\bigl[a+\tilde{b} \epsilon+(n-1)K+a\tilde{L}\bigr]\phi \\ &-\Delta_{f}\phi+2\frac{|\nabla\phi|^{2}}{\phi}. \end{aligned} $$

Substituting the Cauchy inequality

$$\begin{aligned}-\frac{1}{2}\nabla\phi\frac{\nabla h}{h}\leq{}& \frac{1}{2}|\nabla\phi|\frac{|\nabla h|}{h} \\ \leq{}&\frac{n}{4n-3}\frac{|\nabla\phi|^{2}}{\phi} +\frac{4n-3}{16n}\phi \frac{|\nabla h|^{2}}{h^{2}} \\ ={}&\frac{n}{4n-3}\frac{|\nabla\phi|^{2}}{\phi} +\frac{4n-3}{16n}\frac{G}{h^{2}} \end{aligned} $$

into (2.18) gives

2.19 $$ \begin{aligned}[b] \frac{4n-3}{16n}\frac{G}{h^{2}} \leq{}&2\bigl[a+\tilde{b}\epsilon+(n-1)K+a\tilde {L}\bigr]\phi-\Delta_{f} \phi +\frac{9n-6}{4n-3}\frac{|\nabla\phi|^{2}}{\phi} \\ \leq{}&2\bigl[a+\tilde{b}\epsilon+(n-1)K+a\tilde{L}\bigr]+\frac {C_{1}[(n-1)K(2R-1)+\beta]}{R}+ \frac{C_{2}}{R^{2}}, \end{aligned} $$

where $C_{1}$, $C_{2}$ are two positive constants depending on *n*. Hence, on $B_{p}(R)$ with $R>1$, it follows from (2.19) that

2.20 $$ \begin{aligned}[b] \frac{4n-3}{16n}G(x)\leq{}& \frac{4n-3}{16n}G(x_{0}) \\ \leq{}&h^{2}(x_{0}) \biggl[2\bigl[a+\tilde{b} \epsilon+(n-1)K+a\tilde{L}\bigr] \\ &+\frac{C_{1}[(n-1)K(2R-1)+\beta]}{R}+\frac{C_{2}}{R^{2}} \biggr]. \end{aligned} $$

In particular, the estimate (2.20) gives

2.21 $$ \begin{aligned} |\nabla u|^{2}\leq{}&C A^{2} \biggl[\max \biggl\{ \frac{4}{5}b+a \biggl(1+ \frac {4}{5}L \biggr),0 \biggr\} +K+\frac{|\beta|+1}{R} \biggr], \end{aligned} $$

which finishes the proof of Theorem 1.1.

### Proof of Theorem 1.3

We define $\tilde{h}=\log u$. Then we have

2.22 $$ \begin{aligned}[b] \Delta\tilde{h}-\nabla f\nabla \tilde{h}&=\Delta_{f}\tilde{h} \\ &=\frac{\Delta_{f}u}{u}-\big|\nabla(\log u)\big|^{2} \\ &=-|\nabla\tilde{h}|^{2}-a\tilde{h}-b, \end{aligned} $$

where, in the last equality of (2.22), we used Eq. (1.3). Using the Bochner formula with respect to the *f*-Laplacian, we have

2.23 $$ \begin{aligned} [b] \frac{1}{2}\Delta_{f}| \nabla\tilde{h}|^{2}={}&\big|\nabla^{2}\tilde{h}\big|^{2}+ \nabla \tilde{h}\nabla\Delta_{f} \tilde{h}+\mathrm{Ric}_{f} (\nabla\tilde {h},\nabla \tilde{h}) \\ \geq{}&\frac{1}{n}(\Delta\tilde{h})^{2}+\nabla \tilde{h}\nabla \Delta_{f} \tilde{h}-(n-1)K|\nabla\tilde{h}|^{2}. \end{aligned} $$

Moreover, by virtue of (2.22), we have

2.24 $$ \begin{aligned}[b] (\Delta\tilde{h})^{2}={}& \bigl({-}|\nabla\tilde{h}|^{2}+\nabla f\nabla\tilde {h}-a\tilde{h}-b \bigr)^{2} \\ ={}&|\nabla\tilde{h}|^{4}-2|\nabla\tilde{h}|^{2}(\nabla f \nabla\tilde {h}-a\tilde{h}-b)+(\nabla f\nabla\tilde{h}-a\tilde{h}-b)^{2}. \end{aligned} $$

If the assumption (1) holds, then (2.24) yields

2.25 $$ \begin{aligned} [b](\Delta\tilde{h})^{2}\geq{}&| \nabla\tilde{h}|^{4}-2\delta|\nabla\tilde {h}|^{4}+(\nabla f \nabla\tilde{h}-a\tilde{h}-b)^{2} \\ \geq{}&(1-2\delta)|\nabla\tilde{h}|^{4}. \end{aligned} $$

On the other hand, if the assumption (2) holds, then (2.24) shows

2.26 $$ \begin{aligned}[b] (\Delta\tilde{h})^{2}\geq{}&| \nabla\tilde{h}|^{4}-(\nabla f\nabla\tilde {h}-a\tilde{h}-b)^{2}+( \nabla f\nabla\tilde{h}-a\tilde{h}-b)^{2} \\ ={}&|\nabla\tilde{h}|^{4} \\ \geq{}&(1-2\delta)|\nabla\tilde{h}|^{4}. \end{aligned} $$

Therefore, in these two cases, we have

2.27 $$ (\Delta\tilde{h})^{2}\geq(1-2\delta)|\nabla \tilde{h}|^{4}, $$

and (2.23) gives

2.28 $$ \begin{aligned} \frac{1}{2}\Delta_{f}| \nabla\tilde{h}|^{2}\geq{}&\frac{1-2\delta}{n}|\nabla \tilde{h}|^{4}- \nabla \tilde{h}\nabla\bigl(|\nabla\tilde{h}|^{2}\bigr)-\bigl[a+(n-1)K \bigr]|\nabla\tilde{h}|^{2}. \end{aligned} $$

Following the proof of Theorem 1.1 line by line, we obtain on $B_{p}(R)$ with $R>1$,

2.29 $$ |\nabla\tilde{h}|^{2}\leq\frac{C_{1}(n,\delta,\beta)}{R}+C_{2}(n, \delta)\max \bigl\{ a+(n-1)K,0\bigr\} , $$

where *δ* is taken to zero in the second assumption.

We completed the proof of Theorem 1.3.
